# Neural architectures for stereo vision

**DOI:** 10.1098/rstb.2015.0261

**Published:** 2016-06-19

**Authors:** Andrew J. Parker, Jackson E. T. Smith, Kristine Krug

**Affiliations:** Department of Anatomy, Physiology and Genetics, University of Oxford, Sherrington Building, Parks Road, Oxford OX1 3PT, UK

**Keywords:** binocular vision, pattern recognition, cortical columns

## Abstract

Stereoscopic vision delivers a sense of depth based on binocular information but additionally acts as a mechanism for achieving correspondence between patterns arriving at the left and right eyes. We analyse quantitatively the cortical architecture for stereoscopic vision in two areas of macaque visual cortex. For primary visual cortex V1, the result is consistent with a module that is isotropic in cortical space with a diameter of at least 3 mm in surface extent. This implies that the module for stereo is larger than the repeat distance between ocular dominance columns in V1. By contrast, in the extrastriate cortical area V5/MT, which has a specialized architecture for stereo depth, the module for representation of stereo is about 1 mm in surface extent, so the representation of stereo in V5/MT is more compressed than V1 in terms of neural wiring of the neocortex. The surface extent estimated for stereo in V5/MT is consistent with measurements of its specialized domains for binocular disparity. Within V1, we suggest that long-range horizontal, anatomical connections form functional modules that serve both binocular and monocular pattern recognition: this common function may explain the distortion and disruption of monocular pattern vision observed in amblyopia.

This article is part of the themed issue ‘Vision in our three-dimensional world’.

## 1. Introduction

Binocular stereoscopic vision is wholly dependent on the ability of the nervous system to register the presence of small differences in the exact positioning of visual features on the left and right retinae. These differences are created by the horizontal separation of the eyes in the head. Julesz [1] recognized that binocular stereoscopic vision consists of two distinct processes. There is the familiar use of stereoscopic vision to deliver a sense of depth [2,3], but there is also an initial prior stage. Before the binocular disparities in a pair of images can be transformed into a representation of depth, there must be some assignment of which features in the left image correspond to which features in the right image.

**Figure 1.**
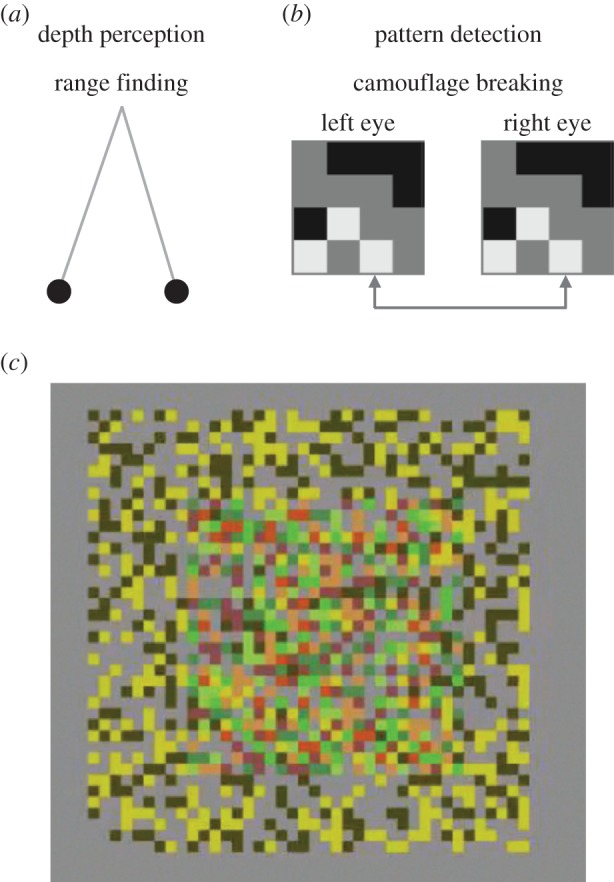
Two processes in stereoscopic vision. The classical view is that stereoscopic vision is concerned with the recovery of binocular depth by triangulation on the small differences in visual images between the left and right retinas (*a*). This cannot proceed to the highest accuracy without precise point-to-point matching of local patterns in the left and right eyes (*b*), which delivers the ability of stereo to break camouflage with a figure–ground separation in depth. (*c*) Both aspects of stereoscopic vision may be viewed with red–green stereo viewing glasses to experience the perception of depth and figure–ground segregation. A dynamic version of this figure is available in the electronic supplementary material.

In figure 1*a,b*, these two processes in stereoscopic vision are illustrated. Triangulation from the eyes (figure 1*a*) can be used to recover the depth of objects in the scene. Figure 1*b* shows the requirement for pattern matching between the left and right eyes’ neural signals. In a crowded set of features with multiple similar features, the brain must find the correct matches between pairs of features (shown by the linking arrow between the left and right eye images in figure 1*b*), otherwise depth cannot be recovered accurately. Julesz highlighted this ‘correspondence problem’, as it became subsequently known [4], with his invention of the random-dot stereogram. Figure 1*c* shows such a stereogram suitable for viewing with red–green stereo glasses.

The relationship that defines the presence of correspondence in stereoscopic vision is essentially structural and mathematical. The formal requirement is to establish the mapping from one field of pattern elements to another. Unless the neural mechanisms can establish this local matching, the brain will be unable to detect any higher-order structural relationships, which in the case of stereo vision reveal the size and shape of the figure defined by the differences in binocular disparity, as well as the figure's depth profile.

Considering the relationship as a mathematical abstraction is useful because it highlights the problem in the form in which it is presented to a network of neurons in the visual cortex. These neurons receive signals from various sources, rearrange the signals according to the connectivity within the network and pass these rearranged signals onward to further neurons. If the connectivity of the network has been set by learning rules or processes of developmental plasticity that are local to the neurons in the network, then, in many ways, the source of the input signals does not really matter. Locally, the neurons just receive signals from multiple sources, respond to the consistent, common components of those signals and then pass the result onward to other neurons. Regarded in this way, there is nothing special or unique about the sources of the signals being processed. Whether or not the common components happen to derive from stereoscopic correspondence is incidental.

Julesz understood the logic behind this point: in his 1960 paper [1, p. 1151], he hypothesizes a ‘binocular pattern recognition [which] we regard as being identical to ordinary (monocular) pattern recognition’. Since that time, a great deal has been learnt about the neural mechanisms underlying binocular stereoscopic vision [5,6]. This paper will analyse the architecture and neural connections of the visual cortex that underlie the establishment of stereoscopic correspondence in binocular vision.

## 2. Neural processing of binocular stereoscopic vision

For isolated visual features, the question of how the nervous system matches features in the left and right eye's images is potentially explicable at the neuronal level, as a result of the discovery of neurons in the primary visual cortex with specific binocular properties [7,8]. These binocular cortical neurons receive input from both left and right eyes via the visual thalamus, with the inputs to the cortex arranged such that the strongest responses of the neuron are produced by visual inputs of similar orientation and contrast polarity presented simultaneously to the left and right eyes. These neurons are also highly sensitive to the exact positioning of the stimulus in the left and right eyes, leading to a potential explanation of the high performance in behavioural measures of stereoscopic acuity [9]. The difference in position in the left and right eyes is termed binocular disparity: across a population of these neurons, it is found that neurons differ in their preferences for the size of binocular disparity, which is equivalent to these neurons being most responsive at different binocular depths [7].

While isolated contours are certainly encountered from time to time in our everyday visual environment, our stereo system must also operate in a different kind of visual scene, which is rich in many overlapping contours and features. Helmholtz noted the ability of stereoscopic vision to contribute to the analysis of these complex binocular scenes, referring to the vividness with which cracks, cavities and surfaces could be resolved in stereoscopic photographs of glacial ice [10]. Julesz was the first to bring this aspect of binocular vision under rigorous experimental study and he was also the first to articulate the need for a computational approach to resolving complex binocular scenes with multiple potential matches, arising from the presence of multiple similar features in the left and right eyes’ images.

If we look in detail at a pair of image regions (one from the left eye, the other from the right) arising from one of Julesz's random-dot figures (figure 1*b*), they consist of a pair of micropatterns with locally corresponding features across the grid of dots. In the construction of these patterns, whether a particular dot is black or white is the outcome of sampling a random process, but once it is determined that a dot in the left eye's image is black, then the corresponding dot in the right eye's image must be black also. Hence, the relationship of the dots when comparing the eyes’ images with each other is fixed rather than random. The pair of arrows linked together in figure 1*b* shows the correct correspondence between a pair of white dots, one from each eye. Julesz pointed out that this pairing must be established in the presence of multiple alternative possible pairings, which in figure 1*b* are available by pairing with one of the other two nearby white dots.

## 3. Visual cortical areas that potentially contribute to stereoscopic processing

**Figure 2.**
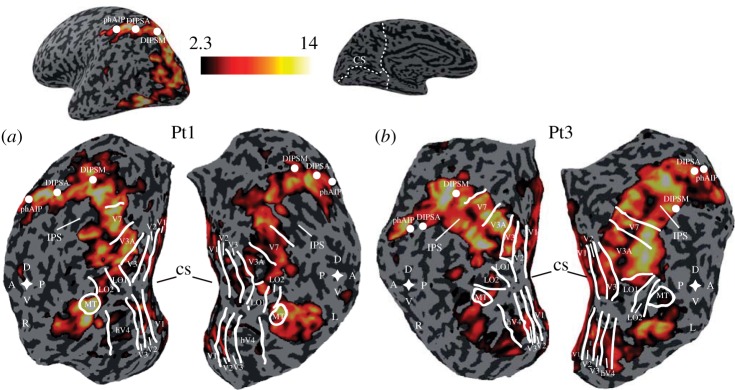
Activation of human cortical responses by stereoscopic depth tasks is widespread, including much of occipital visual cortex and parts of parietal cortex. Heat-map colour scale of *z*-scored activations (two participants in *a* and *b*, respectively) in response to two fields of random dots with a sinusoidal depth profile either side of central fixation point, compared against the response to a flat plane. Subjects performed a task to judge depth of sinusoidal modulation. Anatomical landmarks on the computationally flattened maps of cortex: R and L, right and left cortical hemisphere; D, A, V and P, dorsal, anterior, ventral and posterior axial directions; CS and IPS, calcarine and intraparietal sulci. Functional areas: V1–V7, hV4, LO1, LO2 and MT, visual cortical areas; parietal areas: dorsal intraparietal sulcus anterior (DIPSA), dorsal intraparietal sulcus medial (DIPSM) and putative human anterior intraparietal (phAIP). Reproduced with permission from Minini *et al*. [11].

Neurophysiological studies in visual areas of the macaque monkey have shown the presence of neurons tuned selectively for binocular disparity (see Parker [6] for review). One of the most striking observations about how the cortex responds to binocular depth is the widespread nature of these responses, which cover many identifiable areas of visual cortex and beyond. Human imaging studies also confirm this feature of the cortical response to binocular depth: figure 2 shows an example from such a study [11] that compares the response to stimuli containing stereoscopic depth (sinusoidal spatial modulation of the disparity field) with the response to a stimulus in which all dots lie in a flat zero disparity plane. The activations of cerebral cortex are extensive, including many visual areas and also out from the occipital cortex to parietal regions such as DIPSM, DIPSA and phAIP (see figure legend for definitions).

Nonetheless, there is differentiation among the responses of these multiple cortical regions. The strongest evidence for this comes from neurophysiological studies, conducted in rhesus macaques. Neurons in the macaque extrastriate cortical areas exhibit a range of more advanced stimulus preferences that indicate a transformation of information about binocular depth, as signals move from the primary visual cortex out into extrastriate cortex. The responses of V1 are dominated by simple matching between binocular features from left and right eyes but, beyond V1, neurons acquire new signals: extrastriate neurons carry information about relative depth as well as absolute depth [12–15], they acquire choice probabilities for depth-related visual tasks [16–20] and the neuronal response to binocular anti-correlated depth stimuli is suppressed as signals penetrate into deeper layers of cortical processing [21,22]. All these observations suggest that neurons in visual cortex outside V1 are more closely involved in perceptual judgements about binocular depth. An aim of this paper is to compare the neural architecture for processing of binocular depth within and outside the primary visual cortex.

## 4. Cortical architectures in V1 for binocular vision

When input arrives from the left and right eyes into the primary visual cortex (area 17), the initial locations in which the thalamic axons lie are strongly segregated, with some locations being dominated by the left eye and others by the right eye [23]. The segregation of the thalamic axons by eye of origin is also accompanied by a generalized retinotopic ordering of the projection of thalamic axons across the cortical surface, such that the axonal map aligns with the retinotopic organization of the responses of the cortical neurons themselves. This alignment is sustained by vertical neuronal connections, which are aligned normal to the cortex's multiple layers. This retinotopic organization extends across the entire surface of V1 and the boundary between one retinotopic map and the next defines the separateness of individual cortical areas within visual cortex as a whole. Putting together binocular inputs with retinotopy means the retinotopic map must break regularly to accommodate the axons arriving from each eye. These breaks occur where the cortex switches from being dominated by one eye to being dominated by the other eye, so they mark the boundaries of the ocular dominance (OD) columns.

Hubel and Wiesel first identified these structures, which are especially prominent in layer 4 of V1. They conceived that within a single OD column there might be a small but steady progressive change in the retinotopic location of the neuronal receptive fields across the width of one column. At the column boundary, the neural signals switch to being dominated by the other eye and at the same time the preferred position shifts back, against the previous progression, by about half the spatial distance traversed in the neighbouring column devoted to the other eye [23]. With this sequence of interlocking and ratcheted steps, each eye's input gains a fair share of cortical resources, yet the topographic progression of the retinotopic organization is consistent across the cortical sheet of V1.

The above description was very much derived from work with Old World monkeys, chiefly macaque. Different forms of visual cortical mapping are found in other species [24], with even a vigorous debate about the arrangements in other non-human primates [25]. However, the evidence appears to be that human cortex is similar to that of Old World monkeys [26–28]. This connectivity supports an initial sampling of the inputs from the left and right eyes, which could potentially form the basis of disparity selectivity. For example, a pair of inputs from left and right eyes that lie near a column boundary would be spatially offset by the distance traversed in the topographic map across a single OD column. This offset would be enough to generate a spatial difference in monocular locations for disparity signalling (as in Yeshurun & Schwartz [29]). To generate this limited form of disparity selectivity, it would be sufficient to have a pair of short-range connections from eye-specific neurons in layer 4, running vertically across the cortical layers, to a single neuron in layer 2/3, where many binocular neurons are thought to reside.

This highly local wiring to form binocular neurons mandates a maximum offset between left and right eye signals, which is the size of an OD column projected back out into the spatial world. If the spatial separation of inputs that is required to serve stereoscopic vision proved to be greater than the size of a single OD column, then this suggests that the relevant anatomical connections must be established by lateral neural connections that pass horizontally across the cortex.

The idea under discussion here is that stereoscopic disparity might be processed by neural connectivity structured by the neighbourhood relationships of OD columns [29]. A distinct and separate proposal is that the OD characteristics of individual cortical neurons, including those outside layer 4, are directly linked to their disparity selectivity. In this case, the pattern of a neuron's disparity selectivity would be predictable from monocular measurements of the relative strength of inputs to that neuron from the left and right eyes taken separately. The presence of this link at the level of individual neurons in V1 in macaque has been questioned by a number of quantitative studies [30,31]. It is hard to prove conclusively the absence of such relationships, but for present purposes, it is sufficient to note that the question of a role for OD columns in forming stereoscopic sensitivity is a distinct hypothesis.

Evidence that lateral connections passing horizontally across the cortex may be important for binocular vision is available from the observations with injections of the anterograde tracer biocytin, either targeted into the centre of an OD column or avoiding the centres of these columns [32]. Injections into the centre of an OD column send connections dominantly to the nearest OD column responsive to the same eye and weaker connections further away; injections of biocytin that target sites in the cortex away from the centres of OD columns result in a spread and spatial distribution of tracer similar in form to the monocular injection sites [32], but avoiding the centres of OD columns. It was assumed that injections that avoid the centres of OD columns are targeting binocular sites in V1, so that the neural projections from those sites are also presumably targeting neighbouring binocular sites. However, there were no experimental measures of disparity selectivity or binocular summation available to correlate with the anatomical data. The binocular interconnections appear to have a narrower spread, given that the monocular horizontal connections ‘skip’ over an OD column belonging to the other eye, whereas binocular interconnections appear simply to connect immediately neighbouring sites. Later studies with injections of cholera-toxin B (CTB) show a wider range for all types of lateral connection, up to 6 mm in V1, presumably reflecting improved sensitivity in the methodology [33].

## 5. Measures of interaction distances for stereoscopic vision across the macaque visual system

Most of the above discussion has concentrated around the cortical organization for disparity selectivity in V1. Signalling of disparity by V1 neurons is simply an initial stage in the generation of neuronal responses that can potentially explain the behavioural and perceptual responses to stereoscopic depth [6,34–36]. While the multiple relationships between V1 and the many extrastriate cortical areas potentially offer a large number of comparisons, in this paper we consider the cortical connectivity required to support neuronal responses specific for binocular disparity, comparing the organization of V1 with the extrastriate cortical area V5/MT.

This comparison is potentially revealing as V5/MT neurons possess a range of properties which indicate that this stage of processing is closer to the requirements to explain perceptual behaviour [12,18–20,37–39]. Unlike V1, V5/MT contains neurons with many characteristics that are predictive of performance in binocular depth tasks. Crucially, injection of small amounts of electrical current into the cortical network of V5/MT at functionally identified locations can bias the monkey's decision in binocular depth tasks [39–41]. This result suggests that neurons in V5/MT are located along the causal pathway for the types of binocular depth task explored in these studies. Here, we focus specifically on the lateral range of local, intrinsic cortical connections that must be required to build a representation of stereoscopic disparity in these two distinct cortical areas.

### (a) Cortical area V1

**Figure 3.**
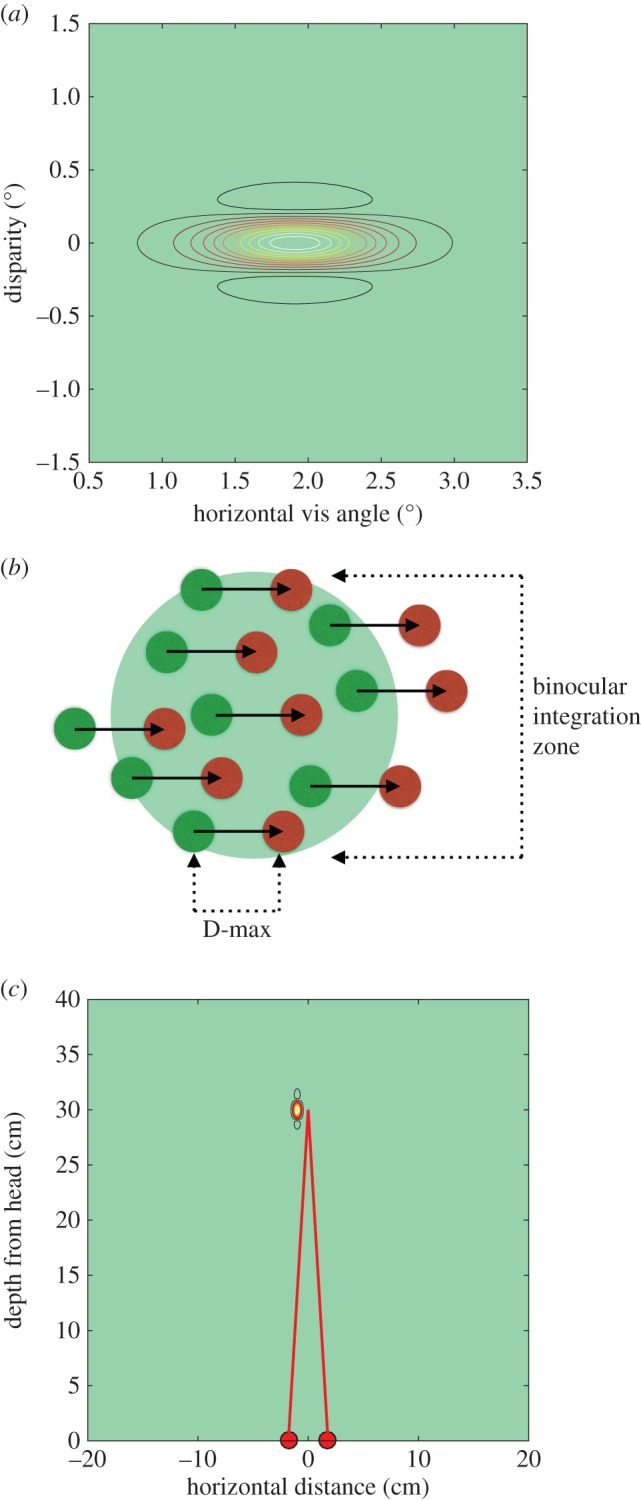
Disparity range and binocular integration zone of a single, disparity selective V1 neuron. (*a*) Contour plot of sensitivity in coordinates of visual angle [30,42] with a heat-map colour coding of neuronal firing rate, as a function of angular disparity shown on the vertical axis and angular lateral extent of binocular integration zone shown on horizontal axis. (*b*) Schematic of binocular response field of the same neuron. Neural activity from points in the left and the right eyes (green and red dots, respectively) arrives within the binocular receptive field of the neuron (large pale green disc). The horizontal separation of the green and red dots represents horizontal binocular disparity, indicated by the length of the black horizontal arrows. The largest disparity that the neuron can encode is here termed D-max. Note that the mid-point of each arrow falls within the binocular integration zone. The neuron integrates signals about horizontal disparity across the binocular integration zone, whose size is shown to the right. (*c*) Same receptive field from (*a*) plotted in terms of real distance referenced to the monkey's head. Red filled circles show position of the eyes and red lines show the optical lines of sight to fixation point, 30 cm from eyes.

For cortical area V1, we need to establish the relevant range over which binocular disparities are computed, which may be derived from earlier work from our laboratory [30,42]. Figure 3 shows a contour sensitivity plot of a single, disparity-selective neuron recorded in V1, together with a schematic interpretation of the binocular, disparity-sensitive receptive field of the neuron. The same receptive field is represented in two ways. In the uppermost panel, figure 3*a*, the receptive field is drawn in angular coordinates, whereas in the bottom panel, figure 3*c*, the receptive field is drawn in spatial coordinates with respect to the position of the monkey's interocular baseline. The latter gives us a scale version of how disparity selective neurons signal the presence of features in the three-dimensional scene before the observer.

To build the picture of how the receptive fields of single neurons relate to cortical architecture, we need to consider how these neurons pool signals for horizontal disparity across their receptive fields. Figure 3*b* introduces a schematic for the interpretation of the quantitative measurements of selectivity for binocular disparity (shown as D-max in figure 3*b*) and the spatial extent over which these disparity-specific responses are pooled (shown as the binocular integration zone). Based upon the analysis of responses in single neurons, we then form a population response for a group of neurons that have been recorded from a distinct subregion of V1. We consider the population response in conjunction with the cortical magnification factor to estimate what these population responses must mean in terms of lateral neural connections across the neocortical surface.

### (b) Integration zone of single V1 neurons for horizontal stereo disparities

The range of disparities encoded by a neuron's receptive field gives us the range of sensitivity to binocular depth, projected back onto the retinotopic coordinate frame of the eyes and the visual cortex. Figure 3*b* gives this schematically. Neural activity from points in the left and the right eyes (green and red dots, respectively) arrive within the binocular receptive field of the neuron (large pale green disc). The horizontal separation of the green and red dots represents binocular disparity and is indicated by the length of the black horizontal arrows. The largest disparity that the neuron can encode is here termed D-max, by analogy with the measure used in human perceptual studies [43,44].

Disparity-sensitive neurons do not simply respond to binocular disparity at a single point in the retinotopic coordinate system. Rather they pool or integrate visual signals over a lateral extent in the same frame of retinotopic coordinates [45]. This is a different dimension that we also need to estimate. The size of this pooling region is termed the binocular integration zone in figure 3*b*. Although this zone extends both horizontally and vertically, it is specifically the integration zone for horizontal binocular disparity. Unlike Cumming [45], there is no consideration here of the range over which vertical disparities are signalled. In terms of figure 3*b*, a vertical disparity would be equivalent to a vertical, rather than a horizontal, offset in the positions of green and red dots representing the inputs from the eyes. It is already established that the binocular integration zone cannot be estimated directly from the receptive field size measured with simple luminance stimuli, such as bars, spots and gratings [46–48]. We need to use stimuli that specifically isolate the disparity-sensitive component of the cortical neuron's response.

**Figure 4.**
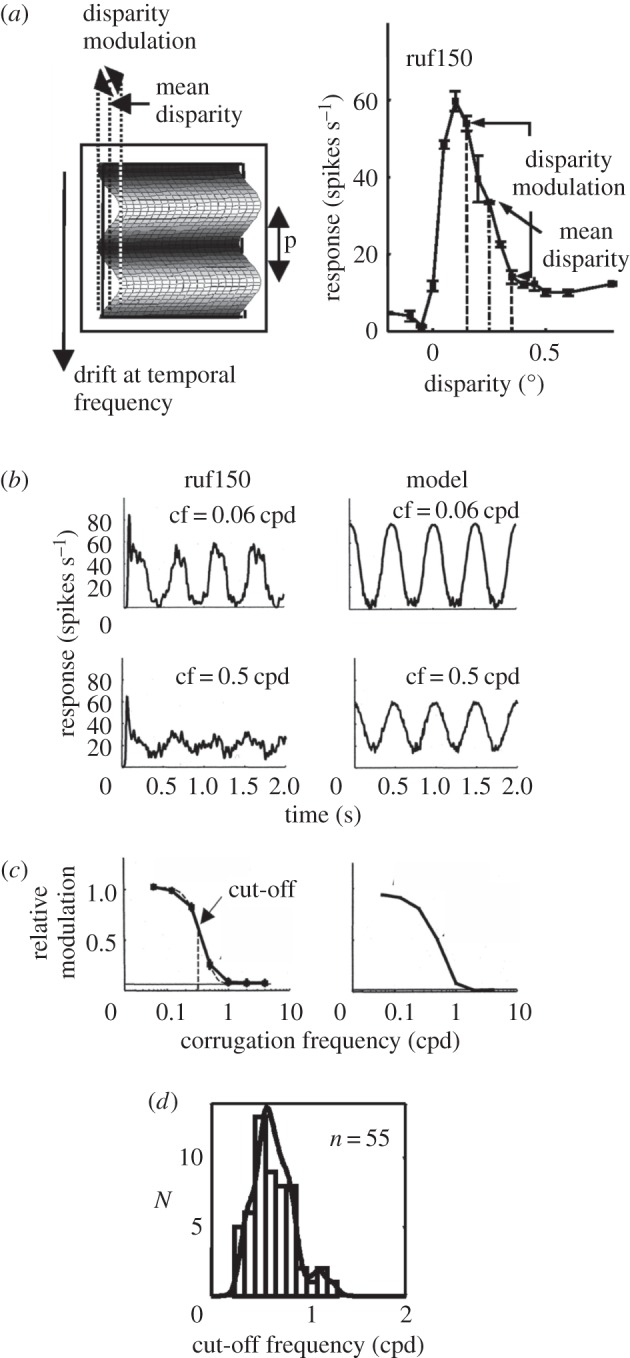
Use of disparity corrugations to estimate the binocular integration zone for horizontal disparity. (*a*) Pattern of dots with sinusoidal modulation of disparity (left) drifts across the receptive field of a neuron (ruf150). Mean disparity and modulation of disparity are selected by measuring the neuron's tuning curve for disparity of planar random-dot stereograms and choosing mean and modulation values of disparity that result in modulation of neuronal firing (right). Various corrugation frequencies were tested for this mean disparity and range of modulation. (*b*) Modulation of neuronal firing (left: ruf150) and response of computational model (right) at corrugation frequencies (cf) of 0.06 and 0.5 cycles per degree (cpd). (*c*) Modulation size decreases as corrugation frequency increases, so that a cut-off frequency for sinusoidal modulation can be defined. (*d*) Distribution of cut-off frequencies for 55 neurons recorded in area V1. Adapted from Nienborg *et al*. [46].

The size of the binocular integration zone for pooling of horizontal disparity can be measured quantitatively by testing the cortical neurons with a field of random dots whose disparity varies with a sinusoidal waveform as a function of spatial position [46]. This spatial waveform is arranged to have peak and trough disparities whose difference causes substantial modulation of the neuron's firing rate as the waveform is drifted across the receptive field (figure 4*a*). A roughly sinusoidal modulation of firing rate results, which declines in amplitude as the spatial frequency of the waveform is increased (figure 4*b*). Quantification of the point at which the modulation falls to two-third of its peak is both a measure of the acuity of the neuron for detecting spatial modulation of disparity (figure 4*c*) and a measure of the size of the integration zone over which the neuron gathers information about horizontal disparity [46,48].

The estimates of receptive field integration size for a set of 55 V1 neurons (figure 4*d*) were interpreted using a single Gaussian weighting function (as in figure 4*c*) to describe the size and shape of the binocular integration zone. Use of a single Gaussian is acceptable, given that almost all measurements of the response of neurons to disparity modulation give low-pass, rather than band-pass, tuning curves as a function of disparity modulation frequency (similar to those shown in figure 4*c*). The average high-frequency cut-off for the disparity modulation was 0.5 cycles per degree (figure 4*d*), much lower than the high-frequency cut-off for detection of sinusoidal modulations of luminance in the same neurons [46].

### (c) Population response

**Figure 5.**
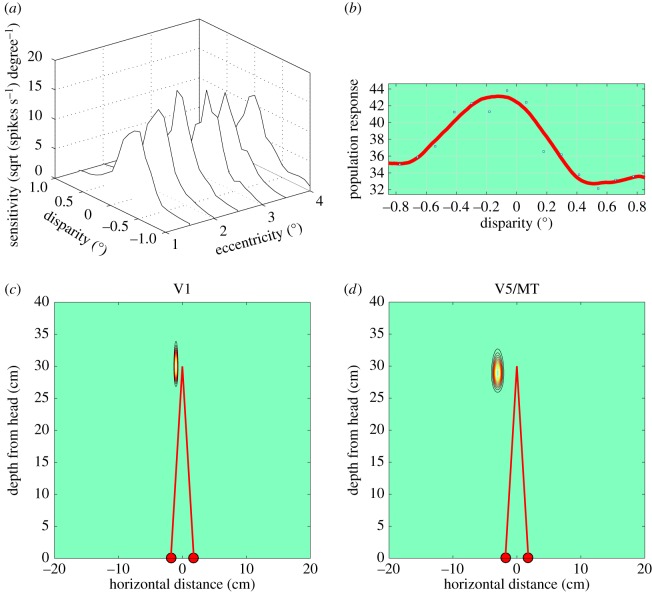
Population sensitivity of neurons recorded in cortical areas V1 and V5/MT. (*a*) Population sensitivity curves from macaque area V1 for horizontal disparity as a function of eccentricity near the fovea, reproduced from Prince *et al*. [30]. (*b*) Population response curve for horizontal disparity in V5/MT, based on pooling responses from 140 neurons recorded in response to planar fields of drifting random dots; data from Krug *et al*. [20]. (*c*) Population sensitivity for V1 neurons plotted in coordinates of binocular depth and horizontal size for a viewing distance of 30 cm as in figure 3*c*. (*d*) Population response of V5/MT neurons in same format as *c*. Note the over-representation of binocular depths nearer than the fixation plane, as in DeAngelis & Uka [49].

In order to gain a measure of the capacity of the population to signal stereo disparity, signals from individual neurons are pooled to gain an estimate of the responses of the population to stereo disparities. The range of the population response for stereoscopic disparity, as estimated by Prince *et al.* [30], is shown in figure 5*a* as a function of eccentricities near the fovea. There is not a great deal of variation as a function of eccentricity over this range, so the population response can be approximated by a single Gaussian curve with a spread (*σ*) of 0.5°. For the dimension of depth away from the head, a Gaussian function with this spread is plotted in figure 5*c*, after being transformed into coordinates of spatial distance relative to the interocular baseline.

This can be combined with the population estimate of the binocular integration zone for stereoscopic disparity. This is derived from the average high-frequency cut-off for disparity modulation in V1 neurons as shown in figure 4*d*. In terms of a Gaussian weighting, this is equivalent to a standard deviation (*σ*) of 0.32°. The schematic of the V1 population response for eccentricities in the range 1–4° is shown in spatial coordinates in figure 5*c*. In total, this is the product of two Gaussian functions, one describing the range of disparities encoded by V1 neurons (from figure 5*a*) and the other describing the binocular integration zone (from disparity modulation measures in figure 4*d*).

### (d) Magnification factor in macaque V1

In order to estimate the cortical distance that is implied by interactions on this scale in visual field coordinates, it is necessary to make use of measurements of the cortical magnification factor: in linear terms, this is the distance in mm across the cortical surface that is devoted to the analysis of 1° of visual angle within the visual field. The magnification factor of macaques and humans varies substantially across the visual field, reflecting the dominance of foveal central vision in the primate visual system. Numerically, magnification factor is large in central foveal vision and declines away from the fovea.

Gaining a quantitative estimate of the cortical magnification factor is not straightforward. Measurements within the foveal region are hard to achieve and published data provide some divergence of estimates. For juvenile monkeys (*Macaca fascicularis*), an early estimate [50] indicated values of 3 mm degree^−1^ at an eccentricity of 2.5°, the closest position with respect to the fovea at which estimates were reliable. This work was based on electrophysiological recording of cells sequentially with a microelectrode and subsequent reconstruction of electrode tracks from histological material.

As an alternative, the visual cortical areas have been mapped in anaesthetized macaque monkeys with measurements of cortical activations using magnetic resonance imaging [51]. These data suggest a somewhat finer representation of the central part of the visual field than the earlier work, with the linear dimensions devoted to the central 11° of the visual field being 25% greater than in Van Essen *et al*. [50]. The difficulty of measuring the magnification factor in the centre of the visual field has been highlighted in human imaging work [52].

The general view is that the representation of primary visual cortex in the macaque is coarser by a factor of two in comparison with estimates from human imaging data [51]. However, the region close to the centre of the field, at eccentricities less than 2.5°, is more uncertain. Accurate measurement in this area requires highly cooperative subjects who can hold their fixation well, so that eye movements do not blur the measurement of the neural image. Accurate measurements also need a high signal-to-noise ratio from the imaging data, which can be hard to achieve with the submillimetre voxel sizes that are ideally needed for measurements in visual cortex. Critically, the retinotopic organization of the cortical maps for visual areas V1–V3 all converge anatomically near this region. A correct assignment of this part of cortex to each visual area needs to be achieved before any meaningful measures of magnification factor may be extracted. The importance of this region to the assessment of binocular vision is apparent from the fact that some 30% of the total area of human V1 is dedicated to analysing the visual signals from the central 3° of the visual field [52].

The measurements of magnification factor in humans that reach into the central 3° suggest values that rise steadily from about 6.5 mm degree^−1^ at 3° to 13 mm degree^−1^ at 0.75°. The extrapolation of the curve fitting the early macaque data fits neatly under this line, with the macaque data being approximately twice as coarse as the human data, much as earlier work suggested [52–54]. Using this approach gives a range of values between 6.5 and 2.5 mm degree^−1^ for the macaque cortical magnification for eccentricities from 1.5° to 4.0°, respectively, with the caveat that these figures could be 25% greater in monkeys of the size and weight used for the disparity tuning measurements. As argued later in the paper, a conservative estimate of the magnification factor is sufficient to sustain the conclusions developed here, so for now we adopt a canonical value of 3 mm degree^−1^ for the region of V1 from which the recordings were made (shown in figures 3–5).

This scaling factor can be applied to the measures of disparity range and binocular integration zone. If a neuron receiving input from one eye at a particular location is to communicate with other neurons receiving input from the other eye, then this may require connections that deliver up to 0.5° of visual angle in either direction (to build neurons selective at the extremes of the V1 disparity range). This implies lateral connections in the cortex of 1.5 mm, reasonably consistent with estimates for V1 derived from anterograde tracing with biocytin [55] and well within the range for later studies with CTB [33]. The lateral connectivity for horizontal disparity is considerably greater than the canonical size of 0.4–0.7 mm for a single OD column in macaque V1 [56]. Some caution is however needed in moving immediately between the measures of receptive field size, cortical magnification factor and size of columnar architecture. It has also been observed that there is variability in the size of OD columns [57], so that a substantial portion of the 0.4–0.7 mm range quoted above arises owing to differences between individual macaques.

A second observation [57,58] is that the size of OD columns is greater in the neighbourhood of the fovea than elsewhere, with again some individual differences between animals. In both cases, individual differences of the same type are manifest in the analysis of histology from the left and right cortical hemispheres of the same animal, suggesting that the measurement and analysis techniques are sufficiently refined to be able to make firm statements. For regions of V1 very close to the vertical midline, there is also an interhemispheric group of callosal connections to be accommodated [59]. These appear to extend as much as 2 mm from the V1/V2 border in the region of the fovea but this narrows to 1 mm away from the fovea. The expansion of OD columns in the region of the fovea [57,58] might be linked to the presence of these callosal afferents.

**Figure 6.**
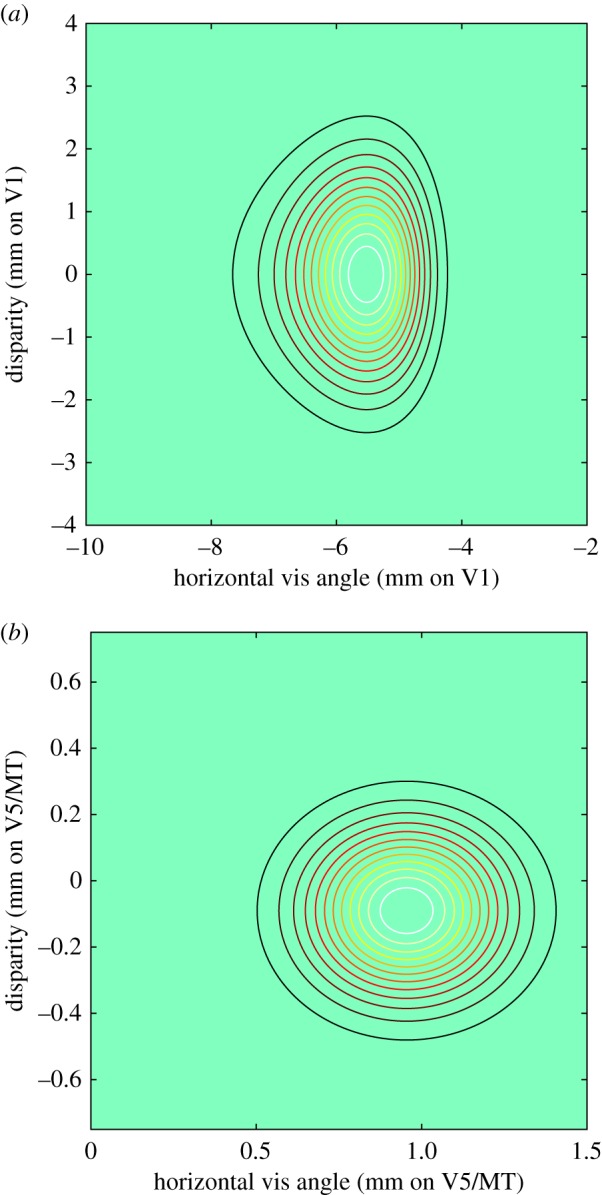
Population sensitivity for disparity from neurons in V1 and V5/MT referenced to units of cortical distance across the surface of each cortical area. (*a*) Sensitivity for V1 population from figure 5*a* transformed by cortical magnification factor from Brewer *et al*. [51]. (*b*) Sensitivity for V5/MT population from figure 5*b* transformed by the cortical magnification factor of 0.5 mm degree^−1^ derived from recording data and analysed in figure 7. Note the different extents of cortical response in comparing *a* and *b*.

Figure 6*a* shows the extent of population sensitivity for stereoscopic vision as laid out on the cortical surface of V1. The contour plot is transformed by the size of the cortical magnification factor from the sensitivity profile in angular coordinates (as shown for a single neuron's receptive field in figure 3*a*). The dimensions in the vertical (*y*-axis) for disparity are derived from the population profiles for disparity range in figure 5*a*. The dimensions in the horizontal (*x*-axis) are the estimated size of the binocular integration zone, summarized schematically in figure 3*b* and measured quantitatively in figure 4*d*.

There are two important conclusions that can be drawn from figure 6*a*.

— First, the disparity range and binocular integration zone are reasonably matched to each other size and are therefore consistent with a roughly isotropic set of horizontal cortical connections running laterally across the surface of V1 [33].
— Second, contrary to Prince *et al*. [30], the disparity range and the binocular integration zone for horizontal disparity are both greater than the size of the OD architecture.

Both these conclusions can be sustained under the conservative assumption that the V1 magnification factor is 3 mm degree^−1^ for the region of the visual field from which the neurons were recorded. If the true magnification factor were larger, then the second conclusion would be more strongly upheld. Note that these conclusions apply to the way in which the cortex processes horizontal disparities: neurons in V1 show a strong degree of specialization for horizontal, rather than vertical, disparity [45].

In summary, the data suggest that a single wiring principle may underlie cortical connectivity for two aspects of stereoscopic vision in V1. There are neural connections that run across visual cortex to build sensitivity to the range of horizontal binocular disparities (shown as D-max in figure 3*b*) encoded within the neuronal population. To deal with the largest horizontal disparities encoded in V1, these connections need to stretch over about 3–4 mm of cortex. Connections running over a similar distance are also responsible for integrating laterally (shown as the binocular integration zone in figure 3*b*), thereby pooling signals about horizontal disparity from nearby horizontal and vertical points in the visual field.

Another way of stating this conclusion is to say that foveal V1 is concerned fundamentally with the representation of space in angular coordinates. Movements in space within the fixation plane and movements in binocular depth orthogonal to that plane traverse the same distance across the cortical surface, when considered in terms of the representation of visual angle in cortical coordinates.

This conclusion is also consistent with earlier findings that the physiological classification of neurons by their OD properties appears to have little significance for the neurons’ responses to binocular disparity [30,31]. The size of the integration zone for binocular stereoscopic vision seems to span multiple hypercolumns, and presumably relies in part upon the network of lateral connections that spread horizontally across the visual cortex. It is interesting to note that the integration size estimated here for binocular vision is about 3 mm, which coincides well with the integration zone of lateral connections estimated from biocytin tracer injections [55] and optical imaging of macaque primary visual cortex [32] with monocular visual stimulation.

### (e) Cortical area V5/MT

The relationship between the scale of visual receptive field properties and visual eccentricity is much shallower in cortical area V5/MT compared with V1. In the macaque, area V5/MT is smaller than V1 by a factor of 10–20× measured in area [51,60,61], such that the entire visual hemi-field is estimated to occupy a region with dimensions between 40 and 150 mm^2^ in extent. However, the three papers quoted all use diverse techniques (single unit mapping, fMRI mapping and histological identification), and there is some divergence in the estimated size of V5/MT, even taking account of differences in body weight and species. It has also been asserted that the mapping of the visual field onto V5/MT may be non-uniform, with an expanded representation of the inferior quadrant in each hemisphere [62]. For these reasons, we decided to re-estimate the cortical magnification factor from our own recordings from V5/MT, as these recordings are most directly relevant for the interpretation of the disparity tuning data obtained during these recordings.

The detailed arrangements for recording from V5/MT used in our laboratory have been previously reported [19,20]. The data presented were recorded from two male macaque monkeys, comprising a dataset of 140 neurons that all showed significant tuning for binocular disparity of both random-dot planar stimuli and rotating cylinder stimuli [20]. The analyses here use only the response to binocularly correlated, planar random-dot stereograms. For each neuron recorded, the location of its receptive field centre in visual space was determined with respect to the animal's fixation point, while at all times monitoring carefully the animal's eye position, including checks on vergence position.

Each neuronal recording site was accessed using an oblique approach to area V5/MT, using a guide tube that was directed towards V5/MT approaching the target from the posterior side. The guide tubes were inserted through a rectangular grid of locations, organized around a reference point defined by the recording chamber that was chronically implanted by surgery at a fixed location on the skull. The verification of the V5/MT recording site was made on the basis of the sequence of grey matter and white matter boundaries encountered during the advance of the electrode, the high proportion of neurons selective for the direction of visual movement, the consistent organization of receptive fields from different recording sites in forming a map and, at the end of the procedure, histological verification.

**Figure 7.**
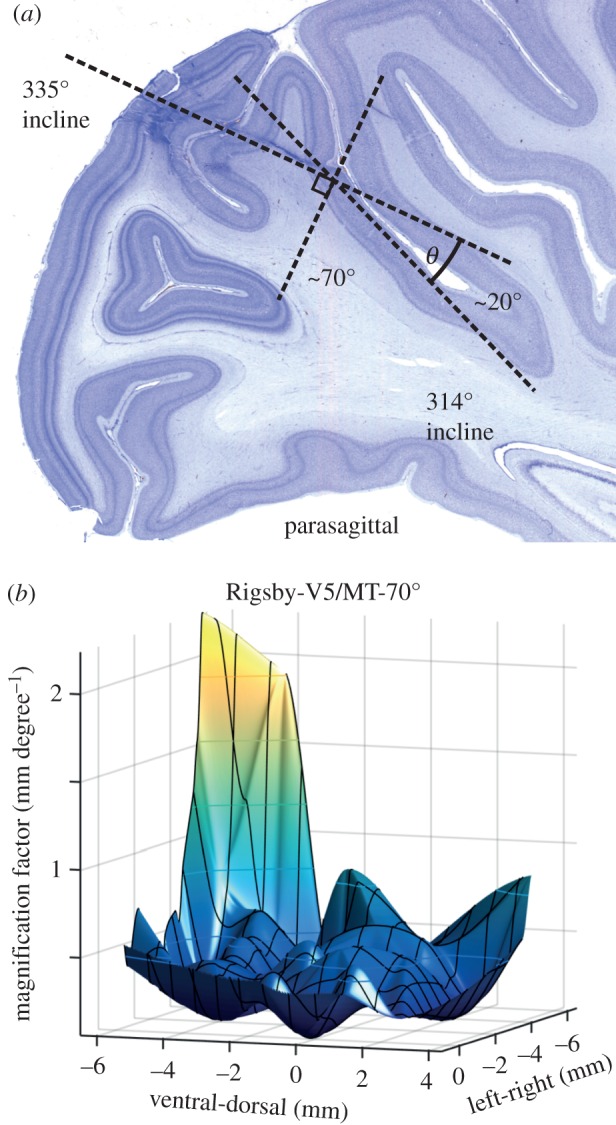
Derivation of local estimate of cortical magnification factor from multiple electrode penetrations into area V5/MT. Entry points of the electrode at the cortical surface were organized on a rectangular grid. The centres of the receptive fields recorded at each grid point were used to estimate how the separation of cortical locations of recording in V5/MT (mm across cortex) related to the separation of receptive fields in visual space (degrees of visual angle) to derive the cortical magnification factor (mm degree^−1^). (*a*) Nissl-stained parasagittal section of macaque cortex, showing the electrode approach to V5/MT on the posterior bank of the superior temporal sulcus and the method for compensating for the angle of approach of the electrodes to the cortical surface of V5/MT. (*b*) Smoothed estimate of cortical magnification factor across grid positions. For further detail of analysis, see electronic supplementary material, figure S1.

An example of histology is shown in figure 7*a*. This shows the location of V5/MT in one macaque monkey on the posterior bank of the superior temporal sulcus [63]. The angle of the cortical layers of area V5/MT with respect to the approaching angle of the electrode (20°) can be seen by reference to the planned orientation of the recording chamber with respect to the cortical surface and the direct measurement from one of the electrode tracks visible in the histology. All other electrode insertions were made within the plane of the cortical section shown in figure 7*a* or in planes parallel to it.

The spacing of these electrode tracks with respect to location across the surface of V5/MT is given by the relative angle between the grid and the cortical surface and the spacing of the insertion points across the grid. There will also be some inevitable variation in the exact trajectory of the electrode, even if it is inserted into precisely the same grid location, because the electrodes themselves may flex or bend a little as they move through the cortical tissue. For these reasons, the analysis of receptive field locations with respect to the cortical surface is based on the averaged value of many estimates of receptive field location from sequential electrode penetrations at the same grid points.

Paired estimates of receptive field locations from different grid positions were referenced to the corresponding pairs of cortical locations in area V5/MT. Each pair of differences contributes one estimate to the calculation of cortical magnification factor (linear distance in mm across the cortex divided by degrees of visual angle separation between the receptive fields). The raw data are shown in electronic supplementary material, figure S1, where they are plotted in cortical coordinates (left–right and dorsal–ventral) and eccentricity in the visual field. The left–right and dorsal–ventral plots confirm the expected topography on the cortical map with higher values of magnification factor towards the fovea being represented as lateral and ventral on the cortical surface. The eccentricity plot shows that cortical magnification factor decreases as eccentricity increases. There is considerable scatter in these data, so a surface smoothing procedure was used to extract the estimates of cortical magnification factor. The resulting values are within the range 0.3–0.7 mm degree^−1^ over most of the area of V5/MT from which recordings were made, with only a few outliers. For the present purposes, we adopt a median estimate of 0.5 mm degree^−1^.

This value of cortical magnification factor was applied to V5/MT measurements in the same way as earlier for V1. First, we took the range of horizontal disparities encoded in V5/MT as plotted in angular coordinates in figure 5*b* and in coordinates of three-dimensional space in figure 5*d*. The data for V5/MT show a slight over-representation of near disparities, as reported previously [49,64], so that the peak sensitivity in terms of depth from the head is slightly nearer than the fixation point in figure 5*d*. It must be acknowledged that there is less certainty about the estimation of the other dimension considered for V1, which is the lateral extent of pooling for horizontal disparity in V5/MT. There are no quantitative estimates based on corrugation sensitivity for V5/MT neurons, as there are for V1 neurons. The V5/MT population response along the axis of horizontal distance as plotted in figure 5*d* is based on the differences in classical receptive field size measurements between V5/MT and V1 [61], under the assumption that the binocular integration zone for horizontal disparity scales proportionately with classical receptive field size between V1 and V5/MT.

In spatial terms, comparing figure 5*c*,*d*, the representation of disparity in V5/MT is somewhat broader than that in the primary visual cortex V1. Figure 6*b* shows the transformation of the population response of figure 5*d* into estimates of cortical distances across V5/MT using the magnification factor derived earlier. Although there is a moderate broadening of the angular size of the population receptive fields between V1 and V5/MT, the representation in cortical distance in figure 6*b* shows that there is actually an anatomical convergence. Thus, based on this analysis, a wide area of V1 anatomically converges to a smaller area of V5/MT (compare figure 6*a*,*b*, noting especially the difference in scales on the axes).

The size of the disparity-processing domain is such that the entire range of disparities can be accommodated within an average distance of 1 mm across the cortical surface of V5/MT. The variation in our estimate of magnification factor might raise this to 1.4 mm or lower it to 0.6 mm at different locations within V5/MT. De Angelis & Newsome [65] conducted a series of recording experiments in V5/MT that identified regions with high disparity sensitivity as the electrode moved through long penetrations of several mm across the V5/MT surface. They concluded that the map of disparity is orderly within these regions. The average size of a module processing both near and far disparities was estimated to be about 1 mm in diameter. The correspondence between that estimate and the one in this paper is further evidence that these disparity domains within V5/MT may comprise a functionally significant level of organization of the cortical architecture.

In comparison with the organization of OD columns in V1, there is of course no exactly equivalent structure for V5/MT, as V5/MT does not possess OD columns. However, anatomical tracing of intrinsic connections in V5/MT does suggest connectivity extending over many millimetres of cortex with repeat patch-like connectivity at approximately 2 mm distances [66]. The functional significance of this repeat distance remains unclear but may be related to conjoint signalling of binocular disparity and direction of motion. Connections on a distance of 2 mm might link domains of cortex that are processing both the same disparity (near or far) and the same direction of visual motion (leftwards rather than rightward motion). Thus, for example, domains processing combinations of leftward motion and near disparities might be specifically connected to each other and similarly for other combinations [66].

## 6. Perceptual measures of interaction distances

It is important to understand whether the measures of interaction distance developed here are applicable to the explanation of perceptual phenomena. With sparse, isolated features, human observers are able to perform a near–far discrimination over wider ranges than maximum range of interaction distance measured here with random-dot patterns. Therefore, the most direct comparison is with perceptual thresholds that are measured with random element stimuli, specifically designed to probe the fine ‘pattern matching’ aspect of stereoscopic vision (figure 1).

For two of the animals from which the V1 recordings were obtained, behavioural tests of their perception are available. Using the dynamic random-dot stereoscopic stimuli employed in the recording experiments, one animal could discriminate between near and far disparities of ±0.6°, whereas the other's range was slightly more limited [30]. This disparity range represents the perceptual D-max [43,44] for the animals, while performing discriminations with dynamic random-dot figures of the same stimulus characteristics as were used for the neuronal recording. There are always potential limitations on the quality of performance working with non-human subjects, so it is reassuring that measurements with human observers resulted in similar upper limits of disparity discrimination [30], although the human data represent the outcome of considerably fewer trials.

## 7. Discussion

On the basis of the measurements of sensitivity to horizontal disparity in cortical area V1, we suggest that V1 is organized for stereoscopic processing into a functional module about 3–4 mm across. This is several times larger than the size of an OD column. The same calculations for V5/MT suggest a domain that is about 1 mm across, in agreement with the estimates for mapping disparity-specific regions of this cortical area [65]. Anatomically, therefore, a spatially extensive region of V1 must send its output to a region of V5/MT that is substantially compressed in terms of cortical coordinates. This is in contrast to the pattern of intrinsic connectivity, where horizontal connections become more extensive in cortical dimensions as one moves further away from V1 [55]. Understanding these relationships is essential for interpreting the density and topography of anatomical projecting fibres that join one region of the cortex to another.

There are further differences in the arrangements for encoding of stereoscopic disparity in these two cortical regions. Within V1, there is a wide range of selectivity for disparity, even locally at the same neurophysiological recording site. When recording with a single electrode, it is possible to extract the activity of single, isolated neurons for comparison with the activity of a cluster of more poorly isolated neurons simultaneously recorded at the same site. In V1, the tuning of single neuron activity for disparity is only weakly related to the tuning of the neighbouring neurons, assessed either in terms of selectivity for disparity or in terms of the preferred disparity of the neuron [42]. This contrasts with the organization of V5/MT for stereoscopic disparity (see fig. 11 in Prince *et al*. [42] for a side by side comparison of V1 and V5/MT). This suggests that encoding for disparity in V1 is embedded within a more broadly specified network that is concerned with encoding other properties of the visual image, as well as disparity. By contrast, the clustering of the domains and the tighter spatial organization of disparity within the cortical architecture of V5/MT is consistent with an important functional role for disparity processing in these domains.

For the case of V1, the data analysed in this paper indicate that the cortical organization for processing of binocular disparity is isotropic, which is a novel aspect that has not been previously identified. We have analysed the disparity range encoded by the population of V1 neurons and the integration zone over which these horizontal binocular disparities are pooled. When these ranges are expressed in terms of cortical representation (scaling retinal angle by the cortical magnification factor), these ranges are matched to one another in cortical distance. We suggest that this reflects a more fundamental feature of the underlying cortical anatomy, which is that the functional module for disparity processing in V1 is defined anatomically by local horizontal connectivity of 3–6 mm in cortical extent [33]. Connectivity on this scale means that each eye-specific, thalamic afferent arriving in layer 4 would exert a direct influence on cortical signalling over this zone of cortex. Clearly, developmental processes such as synaptic plasticity have the capacity to boost or eliminate neuronal connectivity within this isotropic zone. We suggest however that the connectivity identified by Angelucci *et al.* [33] may set an upper limit on the range over which stereoscopic correspondence can be achieved within the V1 network. As such, the anatomical connections that travel horizontally across the cortical surface should be equivalent in extent to the green disc in figure 3*c* that denotes the functional connectivity of the binocular integration zone.

Earlier in the paper, we referred to Julesz’ conception that there might be a binocular pattern recognition system ‘identical to’ the monocular pattern recognition system. We consider further here the idea that this identity between binocular and monocular pattern recognition systems might be fulfilled in part by this highly specialized network of V1 neurons serving foveal vision. Recall that in humans the central 3° of visual field are served by something close to 30% of the primary visual cortex [52]. We examine two different lines of evidence: one from characterizing the consequences of the clinical condition of amblyopia, the other from the disruption of pattern recognition systems by the reversal of contrast signals.

### (a) Amblyopia

Amblyopia is characterized by weaker or disrupted pattern vision, which persists in the absence of obvious pathology in the eye or central nervous system [67]. Amblyopia is strongly associated with abnormal visual experience early in life and is relatively common, affecting some 3–5% of children. Amblyopia is typically monocular, although bilateral amblyopia is recognized clinically. The precipitating factor is the temporary loss of a clear image in one eye, for a variety of reasons ranging from opacity in the eye's optical system or refractive errors of the eye over to wearing of an eye patch during a period of recovering from a trauma to the eye. Another major cause is a deviation of the optic axes of the two eyes, manifest as a strabismus.

An eye that exhibits unilateral amblyopia is often referred to colloquially as a weaker or lazy eye. In animal studies that model amblyopia by imposing a period of unilateral deprivation of input to one eye, the synaptic connections from that eye to cortex are reduced. Orthoptic therapy is often targeted at improving the weaker eye by requiring the child to wear a patch over the stronger eye to give the weaker eye a chance to increase the strength of its connections with the visual brain.

However, the concept of the weaker eye is not the whole story. It has been recognized for a long time that the visual symptoms of amblyopia are not simply described by a veil of reduced visibility over one eye [68]. Even when visual stimuli are readily visible, substantially above the detection threshold, many amblyopes report the appearance of distortions in pattern perception in their weaker eye and can be demonstrated to have poor performance in making discriminations of the spatial configuration of patterns [69,70]. For example, sets of evenly spaced straight lines may have a cracked or distorted appearance and the performance of amblyopes in reading small high-contrast letters is unusually sensitive to the presence of nearby contours [68].

The contribution of typical binocular development to the establishment of accurate monocular pattern vision is highlighted in a large-scale screening of visual function in a group of more than 400 adults who either had measurable amblyopia or exhibited risk factors for its development [71]. This study found that the preservation of binocular function is associated with better monocular pattern vision in the amblyopic eye, as measured by letter-chart acuity or Vernier acuity. Moreover, this association is distinct from any reductions of sensitivity of one eye's input to the cortex. The interesting aspect of amblyopia is that disturbances of binocular vision give rise to problems with monocular form vision.

### (b) Pattern matching in stereoscopic vision

Julesz's pioneering work [1,72] revealed the close relationship between pattern analysis and stereoscopic vision, especially in regard to the ability of stereo vision to break camouflage. There is a range of other neural processes involving fine-scale pattern matching in vision, all of which have the common characteristic that the highest sensitivity is achieved when there is a close match in size, shape and contrast polarity of the pattern elements that need to be matched. Examples other than stereo are short-range apparent motion [73], symmetry [74] and repetition detection [75]. The comparison of these other pattern detection tasks with stereo reveals some intriguing parallels. First, amblyopia disrupts the perception of mirror symmetry [76] and motion perception [77–79]; second, there are some similarities of the effect of contrast inversion on the ability to register correspondence in stereoscopic vision and mirror symmetry (inverted contrast stimuli are often referred to as possessing antisymmetry) [80], although inverted contrast in motion processing leads to reverse phi motion [81].

The disruption of stereo with spatially dense random-dot stereo figures by inversion of the contrast relationship between the dots in the left and right eyes was noted by Julesz [1]. The reader with access to a pair of red–green stereo spectacles can experience this collapse of stereo depth perception for themselves in the electronic supplementary material. The movie displays a dynamic random-dot stereogram that has the normal arrangement of matching dot contrasts in left and right eyes in one time period, followed by a second time period in which the contrast relationship between left and right eyes is inverted. The periods with matched contrast are referred to as binocular correlated (as in figure 1) and those with the inverted relationship as binocularly anti-correlated. There is a loss of stereopsis in the time periods of binocular anti-correlation.

Absence of stereopsis with anti-correlated random-dot figures is most complete when a region with disparities programmed into the anti-correlated random dots is embedded within a surround region that is also anti-correlated [82]. Observers are however able to distinguish between anti-correlated random-dot stimuli and a completely random relationship between dots in the left eye and dots in the right eye (binocular uncorrelation) [83,84], with perceptual reports suggesting a reliance on the appearance of binocular lustre.

If we are looking for an explanation of these results in the responses of neurons tuned to binocular disparity recorded from the visual cortex, then there is a striking discrepancy between the perceptual reports and the responses of neurons in primary visual cortex. In awake, behaving monkeys, Cumming & Parker [85] found that the disparity selectivity of V1 neurons was inverted when they were tested with dynamic anti-correlated random-dot stimuli. Crucially, like humans, the monkeys could not discriminate the depth within these patterns, notwithstanding the fact that the same figures created a disparity-specific response in V1 neurons. This certainly does not fit with the idea of a direct correspondence between responses in a network of V1 neurons and a fully formed stereoscopic percept. Rather it underlines the point that V1 contains a module of neurons specialized for the processing of fine-scale binocular pattern matching, based on similarity between the luminance profiles of features in the left and right eyes. Neurons within a module that is fundamentally sensitive to these luminance profiles will show inverted disparity tuning functions under binocular anti-correlation [86].

Cortical area V5/MT of the macaque has been more strongly associated with the perception of stereoscopic depth. Nonetheless, the neurons of this area show substantial modulations to the disparity content of anti-correlated figures [20]. Moreover, even neurons with a measurable decision-related response in a depth task may show modulation to the equivalent anti-correlated stimulus, implying no simple conjunction of different criteria for the role of the neurons in binocular depth perception [20]. The only cortical area so far discovered whose neurons appear to suppress fully the inverted stereo signal is in temporal cortex [21], although area V4 in the ventral stream appears to suppress the inverted response partially [22], as do the neurons in V1 [85] to a lesser degree. Overall, this suggests that further research into ventral stream function is needed to resolve these questions.

## 8. Summary

This analysis and review has focused on the important role of stereoscopic vision as a detector of patterns that correspond between the left and right eyes. This capacity is crucial for animals or humans to exploit stereo for the breaking of camouflage. The same capacity seems to be lost in a targeted way in some forms of amblyopia and, in a way that is not fully understood, this binocular loss also leads to loss of performance in monocular pattern sensitivity in the amblyopic eye. Stereo and monocular pattern detection appear to share resources even though they are apparently different aspects of pattern vision. What is striking about these various types of visual cognitive capability is that the perception of the spatial relationships is immediate and effortless. While the neural mechanisms underlying these capabilities are initially established in the fine-grain spatial map of the primary visual cortex, it is the relationship between this fine-grain map in V1 and neural architectures of the numerous extrastriate visual areas that is responsible for the full performance of this pattern detecting system.

## Supplementary Material

Supplementary Figure 1

## Data accessibility

The data in this paper are a new analysis based on primary data published elsewhere [19,20,30,46]. Data are available on request from the corresponding author.
