# Supplementary material for: Neural architectures for stereo vision
Source: Philos Trans R Soc Lond B Biol Sci. 2016 Jun 19;371(1697):20150261. doi: 10.1098/rstb.2015.0261 (PMC4901455; doi:10.1098/rstb.2015.0261)
Supplement: Supplementary Figure 1 [file rstb20150261supp1.pdf]

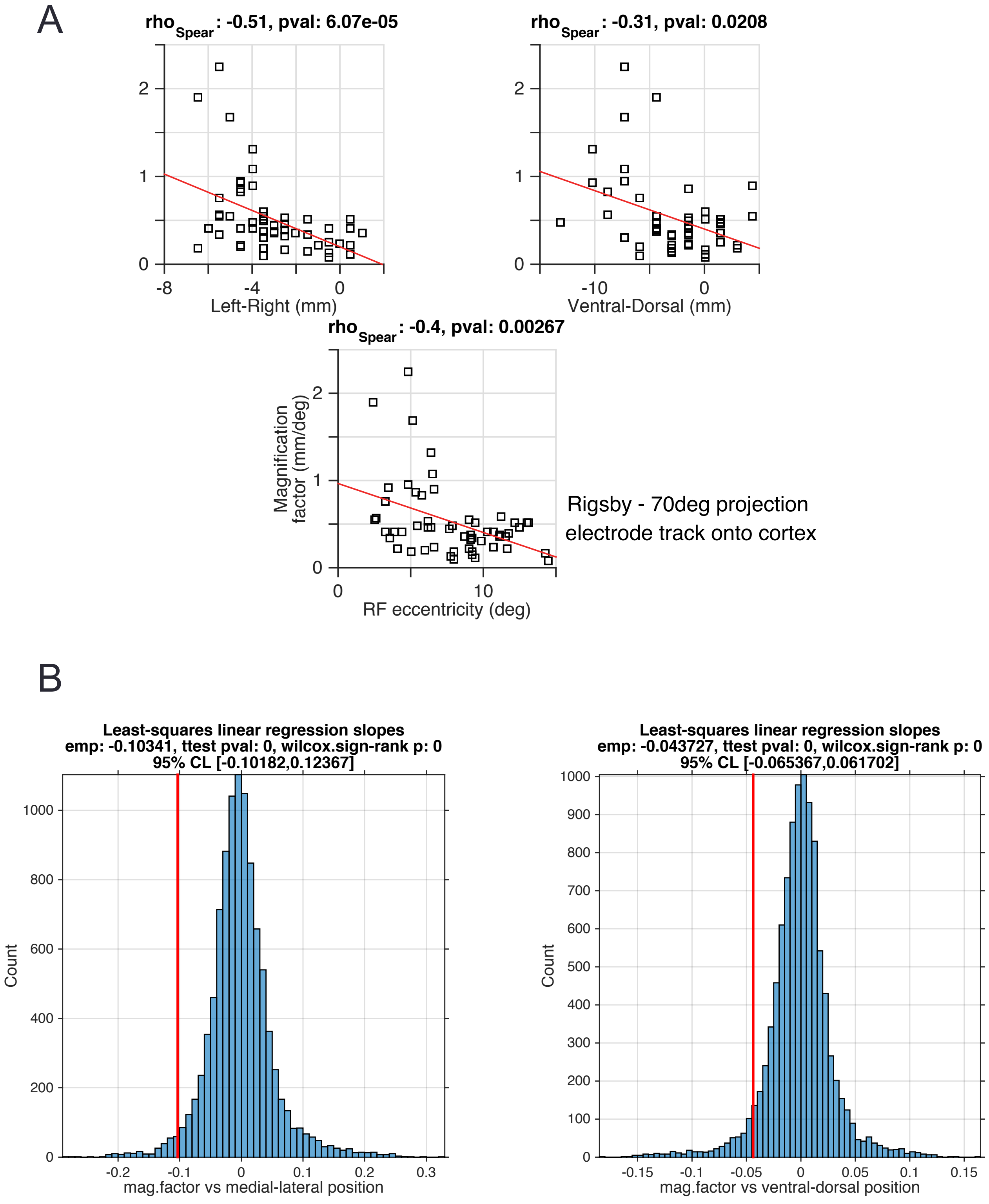

To accompany Parker, AJ, Smith, JET, and Krug, K (2016) Neural Architectures for Stereo Vision. Phil Trans R Soc B, doi 10.1098/rstb.2015.0261

Supplementary Figure 1
